# Prevalence, Molecular Characterization, and Antimicrobial Resistance Profile of Enterotoxigenic *Escherichia coli* Isolates from Pig Farms in China

**DOI:** 10.3390/foods14071188

**Published:** 2025-03-28

**Authors:** Jiajia Zhu, Zewen Liu, Siyi Wang, Ting Gao, Wei Liu, Keli Yang, Fangyan Yuan, Qiong Wu, Chang Li, Rui Guo, Yongxiang Tian, Danna Zhou

**Affiliations:** 1 Institute of Animal Husbandry and Veterinary Medicine, Hubei Academy of Agricultural Sciences, Wuhan 430064, China; xmszjj@hbaas.com (J.Z.); liuzwen2004@hbaas.com (Z.L.); 2023721048@yangtzeu.edu.cn (S.W.); gaoting2017@hbaas.com (T.G.); liuwei@hbaas.com (W.L.); keliy6@hbaas.com (K.Y.); fyyuan@hbaas.com (F.Y.); wuqiong302@hbaas.com (Q.W.); lichang1113@hbaas.com (C.L.); guorui@hbaas.com (R.G.); tyxanbit@hbaas.com (Y.T.); 2 College of Animal Science and Technology, Yangtze University, Jingzhou 434023, China

**Keywords:** Enterotoxigenic *Escherichia coli*, MLST, serotypes, virulence genes, minimum inhibitory concentration, antimicrobial resistance genes

## Abstract

Enterotoxigenic *Escherichia coli* (ETEC) poses a critical threat to livestock health and food safety, particularly in regard to misuse of antimicrobial agents, which have accelerated the evolution of multidrug-resistant (MDR) ETEC strains, reshaping their virulence landscapes and epidemiological trajectories. In this study, 24 ETEC isolates from porcine diarrheal samples undergo genomic and phenotypic profiling, including virulence genotyping, bacterial adhesion, and antimicrobial resistance (AMR) analysis. Results show that multi-locus sequence typing (MLST) outputs (ST88, ST100) and serotypes (O9:H19, O116:H11, O149:H10) exhibited enhanced virulence, with F18ab-fimbriated strains carrying Shiga toxin genes (*stx2A*) demonstrating higher cytotoxicity than non-*stx* strains. There exists a significant negative correlation between bacterial growth rates and intestinal epithelial adhesion, with the expression of ETEC adhesion and virulence genes being growth-time-dependent. These relationships suggest evolutionary trade-offs favoring either rapid proliferation or virulence. Among these isolates, 95.8% were MDR, with alarming resistance to quinolones and aminoglycosides. Geospatial analysis identified region-specific AMR gene clusters, notably *oqxB-aac(3)* co-occurrence networks in 79% of ETEC isolates. These results highlight the urgent need for precision interventions, including vaccines targeting epidemic serotypes and AMR monitoring systems to disrupt resistance propagation across swine production networks. By underscoring the importance of current virulence and AMR profiles, this study provides actionable strategies to mitigate ETEC-associated threats to both animal welfare and meat safety ecosystems.

## 1. Introduction

The domestic pig serves as an important component of the agricultural economy, as over 40% of meat consumption comes from pigs; therefore, the gut health of farm animals is essential for the sustainable development of animal husbandry [1]. However, the intensive use of antimicrobials in commercial swine operations has driven the evolution of multidrug-resistant (MDR) bacterial populations [2,3], which may create critical challenges for food safety and public health through potential transmission along the food chain. Among these emerging threats, enterotoxigenic *Escherichia coli* (ETEC) represents a leading etiological agent of post-weaning diarrhea (PWD), particularly affecting neonatal piglets during critical developmental stages. Epidemiological monitoring has established that porcine ETEC strains express five principal fimbrial adhesins: K88 (F4, including variants K88ac, K88ab, and K88ad), K99 (F5), 987P (F6), and F18 (F18ab and F18ac) [4,5]. Notably, K88-fimbriated strains demonstrate the highest prevalence and significant virulence diversity, frequently co-occurring with K99 or 987P variants in polymicrobial infections [6]. Nowadays, the prevalence of ETEC in swine farms has not yet been studied in the context of the extensive use of antibiotics and vaccines.

Fimbrial proteins, serving as pivotal colonization factors of ETEC, mediate bacterial attachment to intestinal epithelia through specific receptor recognition, thereby initiating pathogenesis [7]. The virulence arsenal of ETEC is further augmented by two principal enterotoxins: heat-stable toxins (STs), inducing cGMP-mediated chloride secretion, and heat-labile toxins (LTs), activating cAMP-dependent fluid loss [8,9]. Once ETEC has adhered to cell surface receptors, ETEC produces these enterotoxins to activate adenylate cyclase (cAMP) and guanylate cyclase (cGMP) [10], resulting in water and electrolyte secretion into the intestinal lumen and causing severe diarrhea, accompanied by high mortality. Therefore, the genotype of fimbriae and toxins are closely related to ETEC infections. Capitalizing on these virulence determinants, contemporary vaccinology strategies increasingly employ structural fimbrial proteins conjugated with toxoids as dual-antigen targets [11,12]. For instance, novel vaccines targeting both ETEC fimbriae and enterotoxins have shown potential efficacy [13,14]. These vaccines aim to enhance protective efficacy, which is especially critical in densely populated swine farming regions [15]. Consequently, systematic surveillance of circulating ETEC genotypes—particularly fimbrial serodiversity and toxin gene combinations—is imperative for implementing precision vaccination programs. Such data-driven strategies not only enhance swine health, but also mitigate zoonotic risks across the meat production continuum, aligning with One Health objectives in sustainable agriculture.

The antimicrobial resistance (AMR) of ETEC is another significant challenge for global food security and One Health sustainability [16]. Intensive antibiotic usage in swine populations has selected for *E. coli* exhibiting pan-drug resistance to ampicillin, tetracyclines, fluoroquinolones, and gentamicin [17,18]. Alarmingly, an increasing number of clinical ETEC isolates now demonstrate resistance to last-resort antibiotics, including colistin, tigecycline, and carbapenems, rendering empirical therapies increasingly ineffective [19,20,21,22]. Another factor, the horizontal transfer of plasmid-borne resistance determinants, also amplifies this health crisis. For example, the plasmid-mediated mobile colistin resistance (*mcr*) gene family encodes phosphoethanolamine transferases, resulting in attenuated affinity for colistin [23]. NDM carbapenemases, which mediate carbapenem resistance, are encoded by the *blaNDM* gene family, such as *bla*_NDM-1_, *bla*_TEM_, *bla*_SHV_, *sul2*, and *aadA* [24], which is harbored by various plasmids, contributing to the transmission of antibiotic resistance [25]. Such prevalence patterns of *E. coli* antimicrobial resistance increase the risk from farming to meat production. Therefore, antimicrobial resistance surveillance plays a crucial role in providing timely therapeutic strategies for resistant infections, promoting the prudent use of antibiotics and mitigating the spread of resistant strains.

To better understand the epidemiological characteristics of ETEC in swine farming, this study isolates and identify ETEC from pig farms across the country. By analyzing the fimbrial genotypes, antimicrobial resistance profiles, and virulence characteristics of these isolates, this study aims to explore clinical strategies to counteract ETEC infections effectively.

## 2. Materials and Methods

### 2.1. Bacterial Isolates

A total of 265 *E. coli* strains were isolated from 83 diarrhea samples obtained from the Xizang, Shanxi, Hubei, Guizhou, and Jiangxi provinces using MacConkey agar (Beijing Land Bridge Technology Co., Ltd., Beijing, China). The specific primers used are presented in Table 1. The PCR reaction mixture contained 12.5 μL of Rapid Taq Master Mix (Vazyme), 0.4 μL of each upstream and downstream primer, and 1 μL of *E. coli* DNA, amounting to a total volume of 25 μL with ddH_2_O. The PCR amplification program consisted of an initial denaturation at 94 °C for 3 min, followed by 30 cycles of 30 s at 94 °C, annealing at 58 °C for 30 s, and extension at 72 °C for 1 min. The PCR products were separated on a 1.5% agarose gel and visualized using a gel imager. The identified ETEC strains were preserved at −80 °C in Luria–Bertani (LB) broth supplemented with 20% glycerol for further study.

### 2.2. Investigation of Virulence

- Hemolytic activity of ETEC strains

The protocol for hemolytic activity was followed according to previous research, although with slight changes [26]. Overnight-cultured ETEC were adjusted to achieve equal bacterial density at a McFarland turbidity of 0.5. A total of 2 μL of ETEC was spotted onto a TSA agar plate with 5% sheep blood (Beijing Land Bridge Technology) and cultured at 37 °C in an incubator. The radius of the hemolytic ring for each strain was recorded after 24 and 48 h.

b. Hemolytic activity of ETEC supernatants

Overnight-cultured ETEC strains were diluted to 10^6^ CFU/mL in LB broth and incubated for 24 h, with the supernatants of the 24 h seed cultures acquired by centrifugation at 10,000 rpm for 5 min at 4 °C and filtered twice through a 0.22 µm membrane filter (vacuum-driven membrane filter, Hi-media). The hemolytic activity of these isolates was measured according to previous research [27].

c. *Galleria mellonella* infection model

The in vivo toxicity of ETEC was assessed in *Galleria mellonella* (*G. mellonella*), which was obtained from Tianjin Huiyude Biotech Company (Tianjin, China) for the infection model. The larvae of *G. mellonella* were randomly divided into groups containing 10 larvae each and infected with 10 μL of ETEC suspension (1.0 × 10^8^ CFU/mL) at the right posterior gastropod region; *Galleria mellonella* larvae injected with 10 μL of PBS served as the negative control. Survival rates of *G. mellonella* were recorded over a period of 5 days.

d. Cytotoxicity

The cytotoxicity assay of ETEC was performed on IPEC-J2 cells (American Type Culture Collection), which were cultured in Dulbecco’s modified Eagle medium (DMEM, Gibco) supplemented with 10% FBS (Invitrogen) and 1% (*w*:*v*) penicillin–streptomycin at 37 °C in a 5% CO_2_ atmosphere. Cells were infected with 500 μL (1.0 × 10^7^ CFU/mL) of ETEC (MOI = 50:1) in 24-well plates and cultured for 3 h; cytotoxicity was scored based on the degree of cell damage under the microscope.

### 2.3. Transcriptional Analysis of Virulence Genes by Quantitative Real-Time qPCR

RT-qPCR was used to analyze the expression of ETEC toxin genes (*faeG*, F:ACGTCGCAGGTTCTTACAGG, R:GCTCCACTGAGTGCTGGTAG; *eltA*, F:TTGGTGATCCGGTGGGAAAC, R:AGGAGGTTTCTGCGTTAGGTG; *eltB*, F:CACGGAGCTCCCCAGACTAT, R:GCCTGCCATCGATTCCGTAT; *estB*, F: TGCCTATGCATCTACACAA, R:CTCCAGCAGTACCATCTC; 16S rRNA, F: CGGTGAATACGTTCYCGG, R: GGWTACCTTGTTACGACTT) at different growth stages. Total RNA was extracted from the ETEC K88 isolate using the EASYspin Plus kit (Aidlab, Hong Kong, China). After measuring the RNA concentration, the qualified RNA was reverse-transcribed using the RT reagent Kit with gDNA eraser (Takara, Kusatsu, Japan). The qPCR procedure was as follows: 95 °C, 5 min; then, 40 cycles of 95 °C for 30 s, 60 °C for 30 s, 72 °C for 30 s. Bacterial RNA of the group that cultured for 0.5 h was used as a control to measure the log2-fold change in gene expression.

### 2.4. Antimicrobial Susceptibility Test

The drug susceptibility of ETEC isolates was measured using the broth microdilution method, as recommended by the Clinical and Laboratory Standards Institute guidelines (CLSI, 2023) [28]. Briefly, twelve types of antibiotics belonging to seven types (Table S3) were diluted twice in Mueller–Hinton broth (MHB, Beijing Land Bridge Technology) and mixed with an equal volume of bacterial liquid (1 × 10^6^ CFU/mL) in a 96-well microtitre plate (Corning). *Escherichia coli* ATCC 25922 was used for quality control. The results were interpreted after 16–18 h at 37 °C of incubation using the CLSI breakpoints.

### 2.5. Library Construction and DNA Sequencing

The bacterial DNA of ETEC strains was extracted using the TIANamp Bacterial DNA Kit (Tiangen Biotech, Beijing, China). The genomic DNA was sent to Personalbio company for sequencing. The total DNA was detected by fluorescent dye (Quant-iT PicoGreen dsDNA Assay Kit), and the genome library was constructed using the standard Illumina TruSeq Nano DNA LT library preparation experimental process. The raw reads were filtered, and high-quality reads (clean reads) were assembled into genome contigs using SPAdes v3.13.0. The bacterial sequences were deposited into the NCBI database (PRJNA1207769).

A phylogenetic tree, including 24 ETEC isolates, O157:H7 (BA000007.3), APEC (NC_008563.1), and K88 (U00096.3 *Escherichia coli* str. K-12 substr. MG1655), was also generated based on the bacterial genome using Harvest v1.1.2 [29]. The tree was rooted in the *Escherichia coli* str. K-12 sequence.

### 2.6. Analysis of AMR Genes, Virulence Genes, and Serogroups

To predict and analyze antibiotic resistance and virulence factors of 24 isolates, the ResFinder and VFDB databases were used for the BLASTn search process [30,31]. The thresholds for gene identity and minimum length were established at a cut-off of 60% of coverage and 80%, respectively. Serotype prediction was performed by building a serotype database based on the SeroTypeFinder (https://cge.food.dtu.dk/services/SerotypeFinder/, accessed on 24 September 2024) of *E. coli*. MLST analysis was conducted using seven conserved housekeeping genes (*adk*, *fumB*, *gyrB*, *icd*, *mdh*, *purA*, and *recA*) according to the *E. coli* MLST database (https://pubmlst.org/data/, accessed on 24 September 2024).

### 2.7. Correlation Analysis

LM was performed using the R software (version 4.1.3) to examine the correlation among bacterial growth, adhesion, hemolytic activity, and cytotoxicity. The co-occurrence networks of ARGs and VFs were generated using Cytoscape v3.8.0 [32].

### 2.8. Statistical Analysis

Statistical analysis was conducted using GraphPad Prism version 9.0. All data are presented as the mean ± SD. A heatmap displaying the distributions of hemolytic activity, growth, adhesion, cytotoxicity, and the prevalence of ARGs and VFs was also generated using GraphPad. The phylogenetic tree was visualized using iTOL v5.

## 3. Results

### 3.1. Characterization and Isolation of ETEC from Different Farms in China

**Figure 1:**
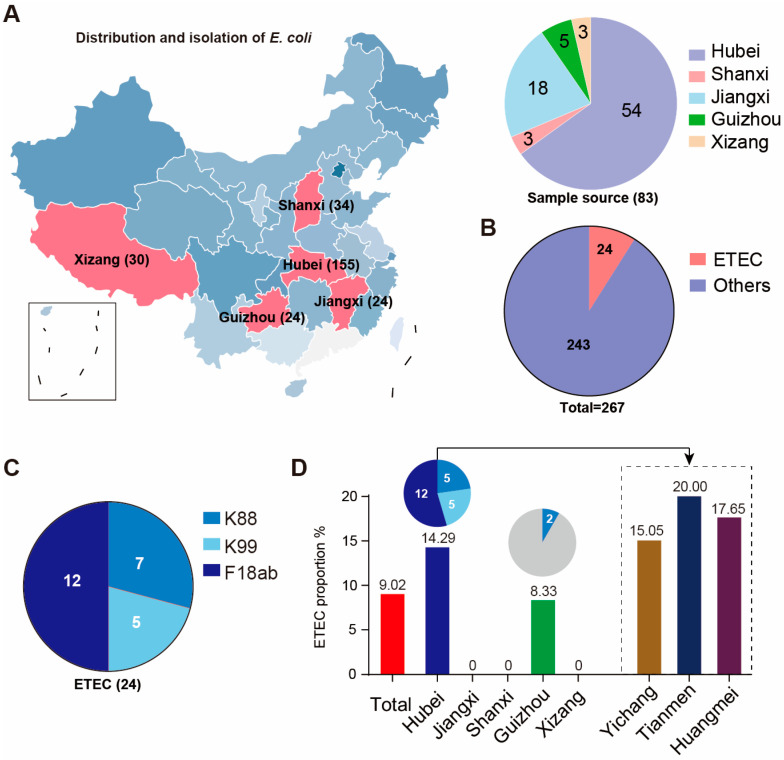
Characterization of ETEC isolates from weaned pigs in China. (**A**) The sample source and distribution of 267 *E. coli* isolates. (**B**) Number of ETEC isolates and total *E. coli* samples. A total of 83 samples from five provinces were collected. (**C**) Bacterial number of K88, K99, and F18ab. Half of the ETEC isolates corresponded to F18ab strains. (**D**) Distribution patterns of ETEC in different provinces. Most ETEC were isolated from Hubei (three cities: Yichang, Tianmen, and Huangmei).

To analyze the current distribution characteristics of enterotoxigenic *Escherichia coli* (ETEC) in piglet diarrhea, 83 diarrheal samples were collected from pig farms across seven cities in five provinces (Figure 1A). A total of 267 *E. coli* strains were isolated from the samples, with 24 of the strains identified as ETEC by multiplex PCR (Figure 1B), including seven K88-positive strains, five K99-positive strains, and 12 F18ab strains (Figure 1C). F18ab isolates accounted for half of the total isolates. The overall isolation rate of ETEC was 9.02% (24/267). The majority of ETEC were from Hubei, with an isolation rate of 14.19% (*n* = 22, 22/155), such isolates being distributed across Yichang (15.05%, 14/93), Tianmen (20.00%, 5/25), and Huangmei (17.65%, 3/17) (Figure 1D). The strains from Yichang were divided into two fimbrial genotypes, K99 and F18ab; Tianmen isolates were of the K88 type, and Huangmei samples carried only F18ab. The ETEC isolation rate in the Guizhou sample was 8.33% (*n* = 2, 2/24) (Figure 1D); notably, two strains of ETEC isolates from Guizhou province were K88-positive.

### 3.2. Evaluation of ETEC Toxicity In Vitro and In Vivo

**Figure 2:**
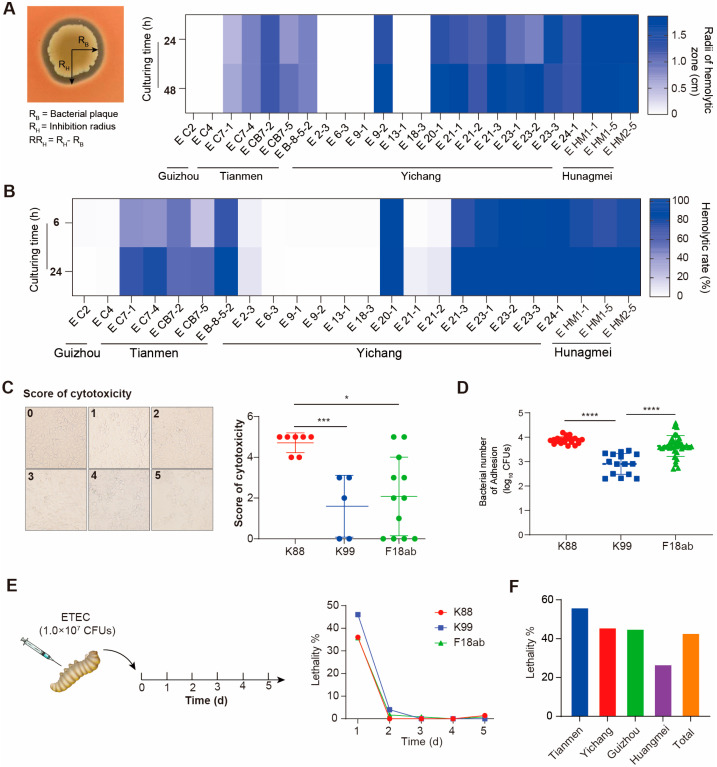
Hemolytic activity and toxicity of ETEC isolates. (**A**,**B**) Hemolytic activity of ETEC isolates and ETEC supernatants. (**C**) A scoring criterion for cytotoxicity and cytotoxicity evaluation of the isolated ETEC. (**D**) Adhesion number of bacteria distributed across K88, K99, F18ab, with a lower adhesion rate of K99 among them. (**E**) A scheme of the experimental protocol for the *G. mellonella* infection model, with the lethality rates of K88, K99, F18ab isolates at a dosage of 1 × 10^9^ CFU/mL (10 μL) injected at the left posterior gastropod region (*n* = 10). K99 strains showed a higher lethality rate than other ETEC strains. (**F**) Lethality rates of ETEC isolates in different regions. ETEC isolated from Tianmen had higher lethality rates than isolates from other regions. Data are presented as the mean ± SD, * *p* < 0.05, *** *p* < 0.001, **** *p* < 0.0001).

We further compared the hemolytic activity and toxicity of ETEC strains with different genotypes. The isolates of the K88 and F18ab strains from Yichang exhibited high levels of hemolytic activity on blood agar plates and in the fermentation broth (Figure 2A,B). However, some strains showed inconsistencies in their hemolytic activity. For instance, the K99 strain E9-2 displayed clear hemolytic zones on blood agar but no hemolytic activity in the fermentation broth. Similarly, strains E21-1 and E21-2 showed reduced hemolytic activity in the fermentation broth despite exhibiting hemolysis on blood agar. Conversely, the E2-3 strain exhibited no hemolysis on blood agar but showed slight hemolytic activity in the fermentation broth.

Then, we conducted a cytotoxicity test to analyze toxins among different genotypes of ETEC by scoring the degree of cell destruction (Figure 2C, left). K88 strains presented higher cytotoxicity on IPEC-J2 cells than K99 and F18ab (Figure 2C, right). Based on the critical factor of ETEC adhesion for toxin synthesis, we evaluated the differences in adhesion to IPEC-J2 cells by counting the number of adherent cells and the growth curve of each isolate. Five K99 strains showed lower adhesion ability than K88 and F18ab (Figure 2D). In particular, the adhesion ability of the 12 ETEC strains varied greatly within the group, while the gap in the K88 group was small (Table S1). The bacterial growth curve showed no significant differences among the ETEC strains (Table S1) and across different fimbrial genotypes (Figure S1).

Further, the *G. mellonella* infection model was used to evaluate the toxicity of ETEC in vivo. The results showed that ETEC caused acute toxicity, with larvae experiencing rapid mortality within the first day post-injection (Figure S2). Among the strains, K99 isolates exhibited the highest mortality rate on the first day, with a 100% lethality rate (Figure 2E). In particular, K88 strains isolated from Tianmen had an above-average lethality rate of 54%, while F18ab isolates from Huangmei showed the lowest lethality rate at 26.7% (Figure 2F).

### 3.3. MLST and Serotype of ETEC Isolates

**Figure 3:**
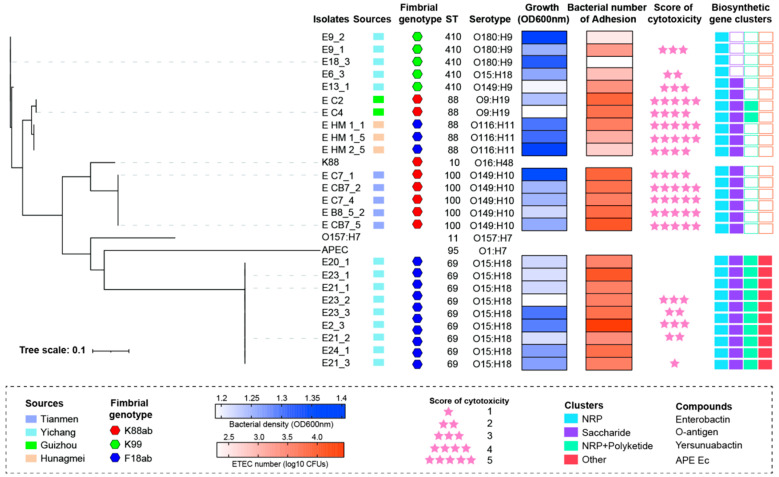
The phylogenetic tree was generated based on the bacterial genome, followed by MLST, serotype, and biosynthetic gene clusters. The tree was visualized using iTOL v5.

MLST and serotype distribution are also methods implemented for bacterial evolutionary and epidemiological analysis. We further analyzed the 24 ETEC strains based on their evolutionary tree. As shown in Figure 3, isolates from the same farm primarily exhibited clonal flow. The 24 strains were classified into four MLST types: all K99 strains were of the ST410 type, K88 strains included ST88 and ST100 types, and F18ab strains were of the ST88 and ST69 types. Interestingly, the same clonal strain was found in different farms; three F18ab strains from Huangmei and two K88 strains from Guizhou were of the same genotype.

With the exception of MLST, there were seven serotypes among these isolates (Table S2). Five K99 strains isolated from Yichang were classified into three serotypes: O180:H9, O15:H18, and O149:H9. The K88 strains also consisted of two serotypes, namely O9:H19 and O149:H10. Among the 12 F18ab strains, three F18ab strains from Huangmei were identified as O116:H11, and the others were identified as O15:H18, the same as K99 E6-3. Next, we predicted the secondary metabolites of the strains by using AntiSMASH and found that all ETEC strains carried NRP gene clusters, which are involved in the synthesis of enterotoxins. Furthermore, except for strains E9-1, E9-2, and E18-3, all other strains carried genes for saccharide synthesis, indicating the potential to produce O-antigens. Lipopolysaccharides (LPSs) consist of lipid A, the core polysaccharide, and the O-antigen. The O-antigen is a polysaccharide located on the outermost layer of the *E. coli* cell wall, playing a key role in determining the serotype, protecting bacteria from host cell phagocytosis, and defending them against complement-mediated killing [33]. These findings underscore the complex interplay of genetic factors contributing to the diversity of ETEC strains.

### 3.4. Toxin Genes Carried in ETEC Isolates

**Figure 4:**
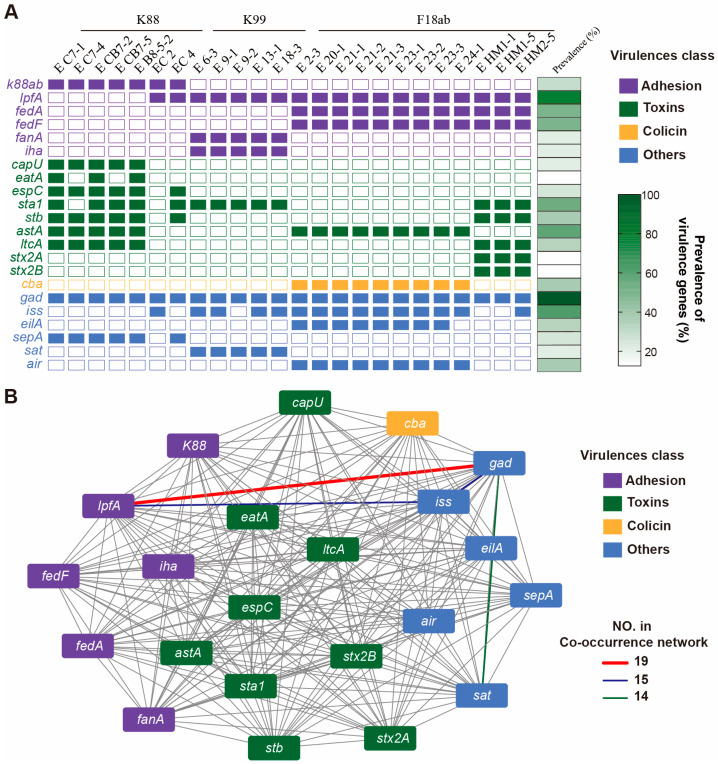
The distribution of virulence genes in the ETEC isolates. (**A**) Virulence genes belong to adhesion, toxins, colicin, and others in the ETEC isolates. (**B**) Co-occurrence network of virulence genes. The adhesion gene *lpfA* and the *gad* gene co-occurred in 19 strains of ETEC, with 15 ETEC carrying *gad* and *iss* genes or *lpfA* and *iss* genes at the same time.

Additionally, the VFDB database was used to analyze the presence of virulence factors in the ETEC isolates. A large number of virulence genes were detected in all ETEC isolates (Table S2). The adhesion genes in ETEC contribute to the synthesis of pili. The K88 strains belonged to the K88ab group, and the F18ab strains carried *fedA* and *fedF* genes. The *fanA* gene was identified as the pilus synthesis gene in K99 strains (Figure 4A). Our results show that K88 isolates carry more toxin genes than K99 and F18ab strains. K88 isolates in this study displayed significantly enriched virulence gene profiles, including *sta1*, *stb*, *eatA*, and *espC*. The K88 isolate EC2 from Guizhou did not carry any virulence genes. Other K88 isolates carried the *espC* gene, which may share a similar DNA fragment with the *espC* gene found in enteropathogenic *E. coli* (EPEC) [34]. Further analysis showed that K99 strains harbor the *sta1* and *sat* virulence genes, and the adhesion-related gene *iha* is commonly found in K99 strains, similar to bacterial iron acquisition proteins [35]. The F18ab isolates from Yichang only carried the *astA* and *cba* virulence genes. Interestingly, three F18ab isolates from Huangmei carried *stx2A* and *stx2B*, which are recognized as enteric shiga-like toxins [36]. Moreover, two Shigella strains were also isolated from the same sample.

Bacterial co-occurrence networks were used to analyze the correlation between two genes. The highest co-occurrence rate (79.2%, 19/24) was found between the *gad* gene and the *lpfA* gene (Figure 4B), followed by a rate of 62.5% between the gad and *iss* genes or between the *lpfA* and *iss* genes, all located in the same ETEC isolate.

### 3.5. Correlation Analysis Among Bacterial Growth, Adhesion, and Cytotoxicity 

Since we observed that strains with faster growth rates had fewer adherent cells, we performed a correlation analysis among bacterial growth, adhesion, and cytotoxicity. The results revealed a significant negative correlation between bacterial growth and the number of adherent cells (Figure S3A). Moreover, the number of adherent cells showed a significant positive correlation with both bacterial hemolytic activity and cytotoxicity (Figure S3B,C).

Subsequently, we extracted bacterial RNA at different growth stages of the strain K88 and analyzed the expression of related virulence genes. Our results indicated that the expression of different virulence genes showed a consistent trend; gene expression gradually increased with the prolongation of culture time and peaked at the stabilization stage (Figure S3D). Considering the correlation between bacterial population and metabolites, we hypothesize that the discovery of a switch that regulates bacterial growth and toxin synthesis could be a target for controlling ETEC infection.

### 3.6. Antimicrobial Resistance of ETEC Isolates

**Figure 5:**
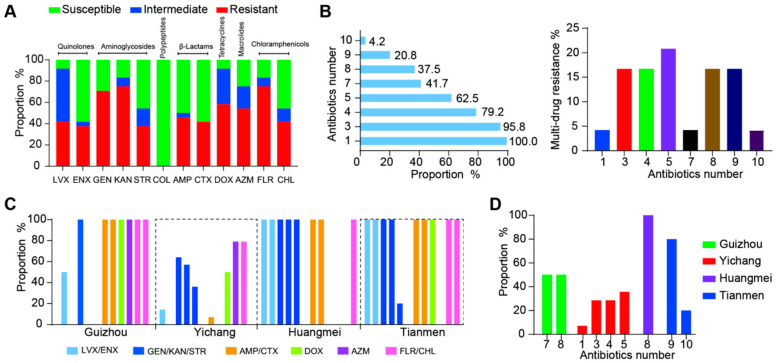
Antimicrobial resistance characteristics of ETEC isolates from pig farms across different provinces of China. (**A**) Histogram illustrating the prevalence of antibiotic resistance in 24 ETEC strains. (**B**) Proportion and bacterial number of multidrug-resistant ETEC strains. (**C**) Prevalence of antibiotic resistance in ETEC strains distributed across different regions. (**D**) Percentages of farm-originating ETEC isolates resistant to different antibiotic classes in different regions.

Given the critical issue of bacterial antimicrobial resistance on farms, we evaluated the resistance characteristics of ETEC strains against 12 antibiotics. The results revealed varying degrees of resistance among all isolates (Table S3). As shown in Figure 5A, over 70% of ETEC strains exhibited high resistance rates to gentamicin (GEN), kanamycin (KAN), and florfenicol (FLO). Resistance rates to levofloxacin (LVX), enrofloxacin (ENX), streptomycin (STR), ampicillin (AMP), cefotaxime (CTX), doxycycline (DOX), azithromycin (AZM), and chloramphenicol (CHL) were 41.7% (10/24), 37.5% (9/24), 37.5% (9/24), 45.8% (11/24), 41.7% (10/24), 58.3% (14/24), 54.2% (13/24), and 41.7% (10/24), respectively. Notably, no resistance to colistin (COL) was observed. Intermediate resistance was detected for LVX and DOX in 50% (12/24) and 33.3% (8/24) of strains, respectively.

Strikingly, 95.8% (23/24) of ETEC isolates displayed multidrug resistance (MDR), with five-drug-resistant strains being the most prevalent (20.8%) (Figure 5B). The prevalence of antimicrobial resistance also varied regionally. ETEC isolates from Yichang exhibited the lowest resistance rates, while isolates from Tianmen had the highest resistance rates (Figure 5C). In particular, Guizhou isolates showed nearly 100% resistance to β-lactams and chloramphenicol, and Huangmei isolates demonstrated nearly 100% resistance to quinolones, aminoglycosides, and β-lactams (Figure 5C). Significant regional differences were also observed in the antibiotic resistance profiles of MDR strains. The Tianmen isolates demonstrated multidrug resistance profiles, with four out of five strains exhibiting resistance to nine antibiotics, while the remaining isolates displayed resistance to ten antibiotics. In Huangmei, all isolates (3/3) were resistant to eight antibiotics. Yichang isolates showed the greatest diversity in MDR patterns, including one-, three-, four-, and five-drug resistance profiles. Meanwhile, the two Guizhou isolates demonstrated resistance to seven and eight antibiotics, respectively (Figure 5D). Alarmingly, the predominance of multidrug-resistant strains within our isolates underscores the escalating limitation of viable therapeutic options, highlighting the urgent need for judicious antibiotic stewardship in swine production systems.

### 3.7. Antibacterial Genes Carried in ETEC Isolates

**Figure 6:**
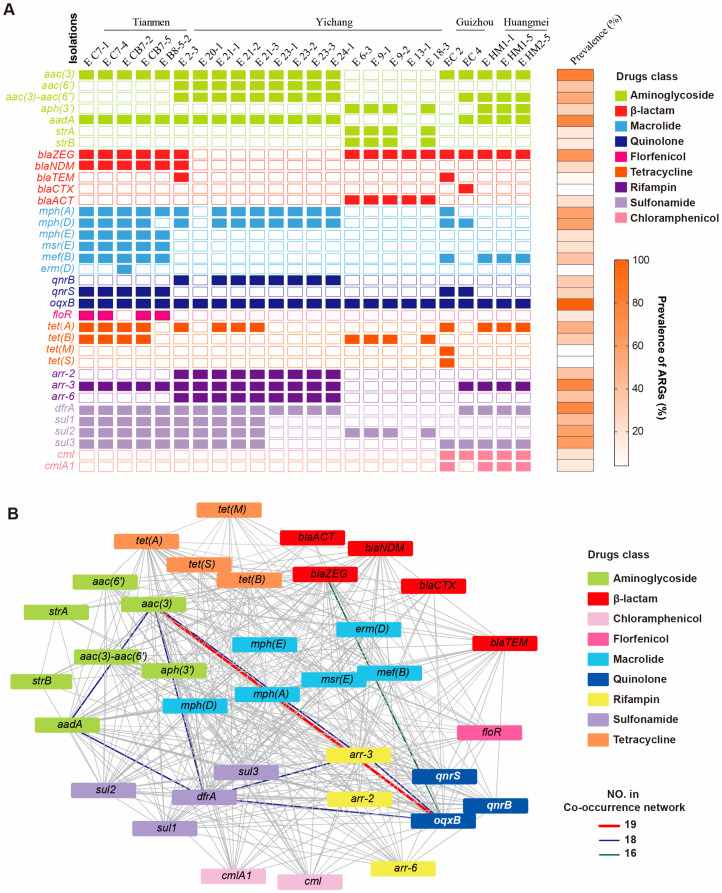
Distribution of antimicrobial resistance genes. (**A**) Distribution of antimicrobial resistance genes in isolates. (**B**) Co-occurrence network of ARGs among the ETEC strains.

Additionally, we analyzed antimicrobial resistance traits in ETEC strains through genetic and phenotypic analysis using resistance gene databases. Thirty-five resistance genes were identified as mediating resistance to nine classes of antibiotics (Figure 6A). All ETEC isolates harbored the *oqxB* gene (100%, 24/24), which is associated with quinolone resistance. This was followed by the aminoglycoside resistance genes *aac(3′)* (79.2%, 19/24) and *aadA* (75%, 18/24), the rifampin resistance gene *arr-3* (75%, 18/24), and the sulfonamide resistance gene *dfrA* (75%, 18/24) (Table S4). Significant regional variations were observed in the distribution of resistance genes among isolates from different farms. For instance, the *qnrB* resistance gene (88.9%, 8/9) was detected exclusively in isolates from Yichang, while *mph(E)* (100%, 5/5) and *msr(E)* (100%, 5/5) genes were only found in isolates from Tianmen. Interestingly, K99 strains carried fewer resistance genes (8/35) compared to K88 (20/35) and F18ab (21/35) strains. Genes such as *strA* (80%, 4/5), *strB* (80%, 4/5), and *blaACT* (100%, 5/5) were exclusively found in K99 strains (Figure 6A). All K88 isolates carried *aac(3)*, *blaZEG*, *qnrS*, and *sul3* resistance genes (100%, 7/7). The *blaNDM* gene was exclusively present in Tianmen isolates (100%). Among the F18ab isolates from Yichang, 100% (9/9) carried the aminoglycoside resistance genes *aac(3)*, *aac(6′)*, *aac(3)-aac(6′)*, *aadA*, and sulfonamide resistance genes. Moreover, significant differences in resistance gene profiles were observed among isolates with different fimbrial genotypes, highlighting the complex genetic basis of antimicrobial resistance in ETEC strains.

Further analysis using a co-occurrence network of resistance genes revealed that the co-occurrence probability of *aac(3)* and *oqxB* was the highest (79.2%, 19/24). This was followed by the co-occurrence of *aac(3)* with *dfrA*, *aadA*, *arr-3*, and *oqxB*, as well as *oqxB* with *aadA*, *arr-3*, and *dfrA*, and *arr-3* with *dfrA*, all of which showed a co-occurrence probability of 75% (18/24). The co-occurrence probability of *blaZEG* with *oqxB* was 66.7% (16/24) (Figure 6B), indicating that these genes may co-transfer during bacterial evolution.

## 4. Discussion

The emergence of enterotoxigenic *Escherichia coli* (ETEC) as a causative agent of post-weaning diarrhea (PWD) and edema disease (ED) in swine populations is intricately linked to its toxin production capabilities and the selective pressures exerted by antibiotic misuse in intensive farming systems [37]. Intensive research efforts are currently focused on developing prevention and control strategies for ETEC, with particular emphasis on polyphenolic compounds [38], probiotics [39,40], and vaccine studies [41]. However, the prevalence of ETEC in Chinese swine farms has not yet been studied in the context of the extensive use of antibiotics and vaccines in recent years. In this study, we focus on the fimbrial genotypes, virulence genes, and antimicrobial resistance profiles of ETEC strains isolated from different farms for the development of actionable strategies to mitigate ETEC-associated threats to both animal welfare and meat safety ecosystems.

Our systematic characterization of 24 ETEC isolates across Chinese swine farms revealed critical shifts in fimbrial genotype prevalence, with F18ab-fimbriated strains dominating 50% of the clinical isolates, a striking contrast to historical reports identifying K88 (F4) and K99 (F5) strains as the predominant adhesins [42]. Regional disparities in fimbrial genotypes were observed among ETEC isolates, with the F18ab variant occurring predominantly in Huangmei. This epidemiological transition may reflect adaptive responses to widespread K88/K99-targeted vaccination programs, which have inadvertently created ecological niches for F18ab-positive clones to thrive. Furthermore, husbandry practices like the use of probiotics, which were isolated from the feces samples, also affect the composition of intestinal flora.

The serotype distribution of the isolates observed in this study demonstrates partial concordance with findings from previous research [43], such as O149 O-antigens, which have been linked to edema disease (ED) in pigs [44,45]. This aligns with the current study, where the K88ab strain isolated from Tianmen had the serotype O149:H10. Notably, the O149:H10 serotype is frequently linked to the K88 fimbrial genotype, and has been recognized as a high-risk clone, similar to O157:H7 in its pathogenicity. Moreover, this serotype has been implicated in piglet diarrhea outbreaks across multiple countries [46,47]. MLST has also proven to be a highly effective method for precise subtype characterization based on genomic variations [48]. Our whole-genome phylogenetic tree revealed that F18ab strains isolated from Huangmei and the K88 strain from Guizhou both belong to ST88. ETEC strains with distinct fimbrial genotypes clustered in closely related phylogenetic branches, suggesting genetic diversity across fimbrial genotypes, sequence types (STs), and serotypes among the isolates. These findings underscore the complex interplay of genetic factors contributing to the diversity of ETEC strains.

Analysis of adhesion and toxin gene profiles in ETEC isolates revealed that highly virulent strains harbor an elevated repertoire of virulence-associated determinants. Our findings demonstrated a direct correlation between adhesion factor expression and bacterial fimbrial genotypes. Notably, K88 isolates in this study displayed significantly enriched virulence gene profiles, including *sta1*, *stb*, *eatA*, and *espC*. The *sta* and *stb* genes encode heat-stable toxins (STs), which are the principal enterotoxins responsible for triggering diarrhea in piglets [49,50,51]. Additionally, EatA, a serine protease autotransporter, facilitates epithelial cell adherence and intestinal colonization by proteolytically processing EtpA, a key exoprotein adhesin [52]. These findings indicate that K88 isolates exhibit markedly stronger virulence potential than strains with other fimbrial genotypes. Intriguingly, F18ab strains carrying *stx2A* and *stx2B* virulence genes demonstrated elevated cytotoxicity and hemolytic activity relative to other F18ab variants. This aligns with the established role of Shiga toxins (Stx) as primary virulence factors in STEC. Such toxins exist in two antigenic forms: Stx1 and Stx2 [53,54]. The Stx2 subtype, particularly Stx2A, is strongly associated with severe clinical manifestations, including hemorrhagic enteritis and hemolytic–uremic syndrome (HUS) [36,55]. Collectively, these results highlight the dynamic horizontal transfer of virulence genes among pathogens, a phenomenon that likely amplifies the pathogenicity of clinical ETEC strains and complicates disease management.

Furthermore, the highest co-occurrence rate was observed between the *lpfA* virulence gene and the *gad* (glutamate decarboxylase) gene. The *gad* gene, which plays a critical role in energy metabolism [56], is directly linked to essential bacterial processes such as growth and toxin production, suggesting its potential indispensability for ETEC survival. In contrast, *lpfA* is a gene associated with ETEC adhesion [57]. This inverse relationship could reflect a trade-off between adhesion capabilities and metabolic adaptation, highlighting the complexity of ETEC virulence strategies. Given that toxin production is typically regulated due to its costly synthesis, competing colonies produce toxins, while fast-growing cells produce non-toxins [58]. Notably, bacterial population dynamics are closely related to bacterial quorum sensing (QS) [59], a cell density-dependent communication system to regulate the expression of virulence factors in *E. coli* [60]. These findings indicate that intervention in bacterial quorum sensing may also serve as a potential strategy for preventing and controlling ETEC infections. However, the precise mechanisms underlying this interplay and the functional interrelationship between these genes remain unclear and warrant further investigation.

Antibiotic resistance poses a significant challenge in ETEC treatment, and *E. coli* has been reported to play a pivotal role in the horizontal gene transfer (HGT) of ARGs, acquiring resistance determinants and disseminating them to other bacterial strains, thereby accelerating the spread of resistance mechanisms within microbial communities [61]. Moreover, the phenotypic antibiotic resistance patterns showed strong concordance with the genomic presence of corresponding resistance determinants. Our results are consistent with prior reports according to which ETEC strains isolated from swine populations demonstrate high resistance rates to gentamicin, kanamycin, and florfenicol, with complete resistance (100%) observed against ampicillin and florfenicol in specific strains [62]. The high level of resistance to gentamicin is likely attributable to its extensive application in managing *E. coli* infections in critical 1–3-day-old piglets [63]. Similarly, florfenicol resistance has emerged as a direct consequence of the chloramphenicol ban, with florfenicol becoming the preferred therapeutic alternative in veterinary practice [64]. Further genomic analysis revealed a total of thirty-five resistance genes across nine antibiotic classes. Among these, aminoglycoside resistance genes were the most abundant, aligning with the observed high resistance to antibiotics like gentamicin. A particularly striking finding was the universal presence (100% prevalence) of the *oqxB* gene, which encodes an efflux pump associated with fluoroquinolone resistance [65]. These transferable ARGs may accelerate the spread of resistance mechanisms within microbial communities.

## 5. Conclusions

Within the One Health framework, characterizing the current epidemiological trends of ETEC is essential for safeguarding the sustainability and productivity of swine farming systems. The heightened prevalence, widespread antibiotic resistance, and augmented virulence of F18ab strains underscore an urgent need for proactive measures by swine producers and veterinary authorities. Furthermore, the evolutionary trajectory of ETEC strains—characterized by escalating resistance and virulence—necessitates sustained surveillance to address their growing threat to animal and public health. A deeper understanding of the intricate interplay between ETEC virulence evolution and resistance development is critical for informing targeted interventions and ensuring effective disease management strategies.

## Figures and Tables

**Table 1:** The PCR primer sequences for identification of ETEC.

Gene Name	Sequences		Length (bp)
K88	F	TGGTAGTATCACTGCAGAT	343
	R	CACTTTCACTGAACCAACT	
K99	F	GCTCGTATTGACTGGTCT	157
	R	CAGCCGTAGTGAATGAAG	
F18ab	F	ATAACTTGGAGCGGGCAGTTA	252
	R	TTGTAAGTAACCGCGTAAGC	
F41	F	TGCTGATTGGACGGAAGGTC	580
	R	GGTTGAAGCACTTTGCCCTG	
987P	F	TTCAGTGGTTACTCGCTCGC	495
	R	AGTGACAGTACCGGCCGTAA	

## Data Availability

All data analyzed during this study are included in this published article. The raw data are available from the corresponding author upon reasonable request.

## Supplementary Materials

The following supporting information can be downloaded at: https://www.mdpi.com/article/10.3390/foods14071188/s1, Table S1: Antimicrobial resistance of 24 ETEC isolates from pig farms; Table S2: Distribution of resistant genes among 24 ETEC isolates from weaned pigs; Table S3: Distribution of virulence genes among 24 ETEC isolates from weaned pigs; Table S4: Distribution of virulence genes among 24 ETEC isolates from weaned pigs. Figure S1: Survival curves after inoculation with 107 CFUs of ETEC isolates; Figure S2: The bacterial adhesion number and growth performance of isolates; Figure S3: Correlation analysis among bacterial growth, adhesion and virulence, and expression of virulence genes.
