# Supplementary material for: Prevalence, Molecular Characterization, and Antimicrobial Resistance Profile of Enterotoxigenic Escherichia coli Isolates from Pig Farms in China
Source: Foods. 2025 Mar 28;14(7):1188. doi: 10.3390/foods14071188 (PMC11989071; doi:10.3390/foods14071188)
Supplement: Supplementary file 1 [file foods-14-01188-s001.zip › foods-3535768-supplementary.pdf]

Prevalence, molecular characterization, and antimicrobial resistance profile of Enterotoxigenic Escherichia coli isolates from Pig Farms in China

Jiajia Zhu[1], Zewen Liu[1], Siyi Wang[1,2], Ting Gao[1], Wei Liu[1], Keli Yang[1], Fangyan Yuan[1], Qiong Wu[1], Chang Li[1], Rui Guo[1], Yongxiang Tian[1] and Danna Zhou[1*]

1 Institute of Animal Husbandry and Veterinary Medicine, Hubei Academy of Agricultural Sciences, Hubei Wuhan 430064, China

2 College of Animal Science and Technology, Yangtze University, Hubei Jingzhou 434023, China

* Correspondence: zdn66@hbaas.com;

Table S1 Antimicrobial resistance of 24 ETEC isolated from pig farms

Isolation		Quinolone		Aminoglycoside			Polypeptide	β-lactam		Tetracycline	Macrolide	Chloramphenicol	
		LVX	ENX	GEN	KAN	STR	COL	AMP	CTX	DOX	AZM	FLR	CHL
Tianmen	E C7-1	16	>32	>32	>64	0.5	0.5	>128	>128	64	16	>32	>128
	E C7-4	16	>32	>32	>64	32	0.5	>128	>128	64	8	>32	>128
	E CB7-2	16	16	>32	>64	>64	0.25	>128	>128	64	16	>32	>128
	E CB7-5	>16	>32	>32	>64	32	1	>128	>128	64	16	>32	>128
	E B8-5-2	16	32	>32	>64	16	0.125	>128	>128	64	8	>32	>128
Yichang	E 2-3	0.5	1	>32	>64	32	0.5	8	<0.25	8	64	32	16
	E 20-1	<0.03	0.06	2	1	16	0.5	8	<0.25	16	32	32	16
	E 21-1	1	0.5	>32	>64	0.5	0.25	16	<0.25	8	>128	32	16
	E 21-2	0.5	2	>32	64	16	0.25	8	<0.25	16	128	32	4
	E 21-3	2	4	>32	>64	32	0.25	8	<0.25	8	62	16	8
	E 23-1	0.5	0.25	>32	32	16	0.5	8	<0.25	8	32	16	8
	E 23-2	1	0.5	>32	>64	8	0.06	8	<0.25	16	16	16	4
	E 23-3	0.5	0.5	>32	32	0.5	0.125	8	<0.25	8	64	>32	4
	E 24-1	0.25	0.5	>32	>64	>64	0.5	8	<0.25	8	16	>32	8
	E 6-3	0.5	0.5	>32	>64	>64	0.25	8	<0.25	8	32	>32	8
	E 9-1	2	2	1	1	>64	0.25	8	<0.25	32	>128	16	8
	E 9-2	0.5	2	4	>64	>64	1	>128	1	64	>128	8	8
	E 13-1	0.03	0.06	2	2	8	0.125	8	<0.25	16	2	16	8
	E 18-3	1	0.5	2	1	>64	0.5	8	<0.25	32	>128	8	8
Guizhou	EC 2-1	0.5	1	2	64	0.25	0.25	>128	>128	32	>128	32	32
	EC 4	1	8	4	64	0.5	0.125	>128	>128	64	>128	32	64
Huangmei	E HM1-1	4	8	>32	>64	64	0.125	>128	4	4	2	8	32
	E HM1-5	4	8	32	>64	64	0.125	>128	4	4	2	8	32

E HM2-5	4	8	>32	>64	128	0.125	>128	8	8	2	8	32
MIC50 (μg/mL)	1	2	>32	>64	32	0.25	8	<0.25	16	32	32	16
MIC90 (μg/mL)	16	>32	>32	>64	>64	0.5	>128	>128	64	>128	>32	>128
Resistant %	41.7	37.5	70.8	75.0	37.5	0.0	45.8	41.7	58.3	54.2	75.0	41.7
Susceptible %	8.3	58.3	29.2	16.7	45.8	100.0	50.0	58.3	8.3	25.0	16.7	45.8
Intermediate %	50.0	4.2	0.00	8.3	16.7	0.0	4.17	0.0	33.3	20.8	8.3	12.5

Note: LVX (Levofloxacin); ENX (Enoxacin); GEN (Gentamicin); KAN (Kanamycin); STR (Streptomycin; COL (Colistin); AMP (Ampicillin); CTX (Cefotaxime); DOX (Doxycycline); AZM (Azithromycin); FLR (Florfenicol); CHL (Chloramphenicol).

Table S2 The bacterial adhesion number and growth performance of isolates

Isolates	Adhesion number (log10 CFU/mL)	Growth performance ($OD600_{nm}$)								
		0	3	6	9	12	15	18	21	24
E C7-1	3.94±0.01	0.10	0.36±0.00	0.78±0.01	0.94±0.03	1.05±0.04	1.16±0.05	1.26±0.05	1.32±0.05	1.34±0.06
E C7-4	3.68±0.04	0.10	0.43±0.03	0.76±0.01	0.89±0.00	0.99±0.00	1.09±0.01	1.18±0.01	1.23±0.01	1.25±0.01
E CB7-2	3.82±0.07	0.10	0.4±0.00	0.74±0.01	0.88±0.01	0.98±0.01	1.09±0.02	1.17±0.02	1.23±0.02	1.25±0.03
E CB7-5	4.14±0.05	0.10	0.35±0.01	0.74±0.02	0.88±0.02	0.99±0.01	1.09±0.03	1.18±0.03	1.24±0.04	1.26±0.04
E B8-5-2	3.88±0.02	0.10	0.39±0.01	0.73±0.02	0.86±0.02	0.96±0.02	1.07±0.02	1.15±0.02	1.21±0.02	1.22±0.02
E2-3	4.48±0.09	0.10	0.42±0.01	0.79±0.01	0.90±0.00	1.06±0.00	1.18±0.11	1.25±0.01	1.29±0.01	1.30±0.01
E6-3	2.9±0.00	0.10	0.36±0.00	0.77±0.01	0.92±0.00	1.02±0.00	1.12±0.00	1.20±0.00	1.24±0.00	1.26±0.01
E9-1	3.28±0.07	0.10	0.36±0.00	0.77±0.00	0.91±0.01	1.01±0.00	1.11±0.01	1.19±0.01	1.24±0.01	1.25±0.01
E9-2	2.75±0.26	0.10	0.38±0.01	0.86±0.05	1.01±0.06	1.13±0.07	1.24±0.07	1.33±0.07	1.38±0.06	1.40±0.06
E13-1	3.38±0.07	0.10	0.52±0.02	0.52±0.02	0.81±0.02	0.93±0.02	1.05±0.02	1.15±0.03	1.22±0.03	1.23±0.03
E18-3	2.31±0.01	0.10	0.37±0.00	0.81±0.03	0.96±0.03	1.07±0.03	1.17±0.04	1.26±0.04	1.30±0.04	1.32±0.05
E20-1	3.58±0.01	0.10	0.35±0.01	0.76±0.01	0.85±0.01	0.96±0.01	1.10±0.02	1.18±0.02	1.22±0.02	1.22±0.02
E21-1	3.64±0.08	0.10	0.38±0.01	0.78±0.01	0.87±0.01	0.98±0.01	1.11±0.02	1.18±0.02	1.21±0.02	1.22±0.02
E21-2	3.48±0.10	0.10	0.38±0.02	0.79±0.01	0.88±0.02	0.99±0.03	1.11±0.03	1.18±0.03	1.21±0.03	1.22±0.03
E21-3	3.62±0.01	0.10	0.45±0.02	0.80±0.02	0.91±0.04	1.04±0.05	1.17±0.04	1.23±0.04	1.26±0.04	1.27±0.03
E23-1	4.1±0.01	0.10	0.39±0.01	0.79±0.01	0.87±0.02	0.98±0.02	1.11±0.01	1.18±0.01	1.21±0.01	1.22±0.01
E23-2	3.6±0.07	0.10	0.36±0.01	0.76±0.01	0.84±0.01	0.93±0.01	1.06±0.01	1.14±0.01	1.18±0.02	1.19±0.02
E23-3	3.87±0.09	0.10	0.45±0.01	0.83±0.03	0.95±0.03	1.07±0.03	1.19±0.02	1.25±0.01	1.29±0.00	1.30±0.00
E24-1	3.89±0.08	0.10	0.34±0.00	0.77±0.01	0.86±0.01	1.03±0.02	1.18±0.01	1.24±0.01	1.27±0.02	1.27±0.02
EC2	3.94±0.01	0.10	0.36±0.03	0.81±0.03	0.95±0.04	1.05±0.04	1.14±0.04	1.20±0.04	1.24±0.04	1.24±0.04
EC4	3.79±0.04	0.10	0.35±0.01	0.78±0.02	0.91±0.03	1.01±0.03	1.10±0.03	1.16±0.03	1.19±0.03	1.19±0.03
E HM1-1	3.69±0.08	0.10	0.38±0.02	0.75±0.01	0.90±0.02	1.02±0.03	1.12±0.03	1.21±0.04	1.27±0.04	1.30±0.04

E HM2-5	2.9 ± 0.1	0.10	0.39 ± 0.02	0.77 ± 0.01	0.94 ± 0.01	1.09 ± 0.03	1.21 ± 0.04	1.30 ± 0.04	1.36 ± 0.04	1.39 ± 0.04
E HM1-5	3.10 ± 0.09	0.10	0.37 ± 0.01	0.74 ± 0.02	0.90 ± 0.03	1.02 ± 0.04	1.13 ± 0.05	1.21 ± 0.05	1.28 ± 0.05	1.30 ± 0.05

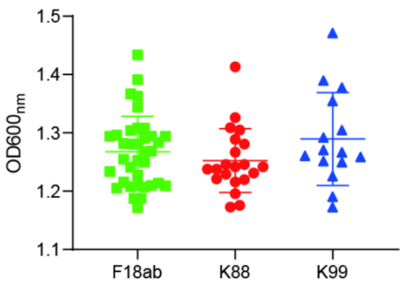

Figure S1 The $OD600_{nm}$ values of different fimbrial ETEC strains after cultured for 24 h.

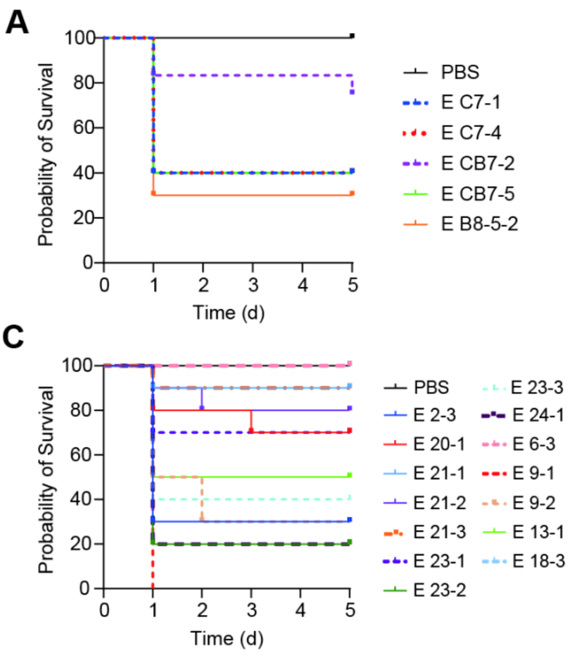

Figure S2 The survival curves of after inoculated ETEC isolates

Table S3 Distribution of virulence genes among 24 ETEC from weaned pigs

VRGs	Product	ETEC (n=24)	Fimbrial genotype			Serotype					
			K88 (n=7)	K99 (n=5)	F18ab (n=12)	O116:H11 (n=3)	O9:H19 (n=2)	O180:H9 (n=3)	O15:H18 (n=10)	O149:H10 (n=5)	O149:H9 (n=1)
**Survival assistant genes**											
gad	Glutamate decarboxylase	24	7	5	12	3	2	3	10	5	1
iss	Increased serum survival	15	1	4	10	1	1	3	10	0	0
**Transcriptional regulators**											
eilA	Regulation factor	8	0	0	8	0	0	0	8	0	0
**Adhesion genes**											
K88ab	Fimbi protein	7	7	0	0	0	2	0	0	5	0
capU		5	5	0	0	0	0	0	0	5	0
lpfA	Long polar fimbriae	19	2	5	12	3	2	3	10	0	1
air	Adherence protein	9	0	0	9	0	0	0	9	0	0
fedA	Fimbi subtype A	12	0	0	12	3	0	0	9	0	0
fedF	Fimbi subtype F	12	0	0	12	3	0	0	9	0	0
iha	Adherence protein	5	0	5	0	0	0	3	1	0	1
fanA	Fimbi protein	5	0	5	0	0	0	3	1	0	1

**Toxin genes**											
eatA	Transportation protein	3	3	0	0	0	0	0	0	3	0
sepA	Serine proteases	6	6	0	0	0	1	0	0	5	0
espC	Extracellular serine protease	6	6	0	0	0	1	0	0	5	0
sta1	Heat-stable toxin STa	13	5	5	3	3	1	3	1	4	1
stb	Heat-stable toxin STb	9	6	0	3	3	1	0	0	5	0
astA	Heat-stable toxin (EAST1)	14	5	0	9	0	0	0	9	5	0
cba	Colicin B	9	0	0	9	0	0	0	9	0	0
sat	Serine protease toxin	5	0	5	0	0	0	3	1	0	1
stx2A	Shiga toxin 2 subtype A	3	0	0	3	3	0	0	0	0	0
stx2B	Shiga toxin 2 subtype B	3	0	0	3	3	0	0	0	0	0
ltcA	Enterotoxin	8	5	0	3	3	0	0	0	5	0

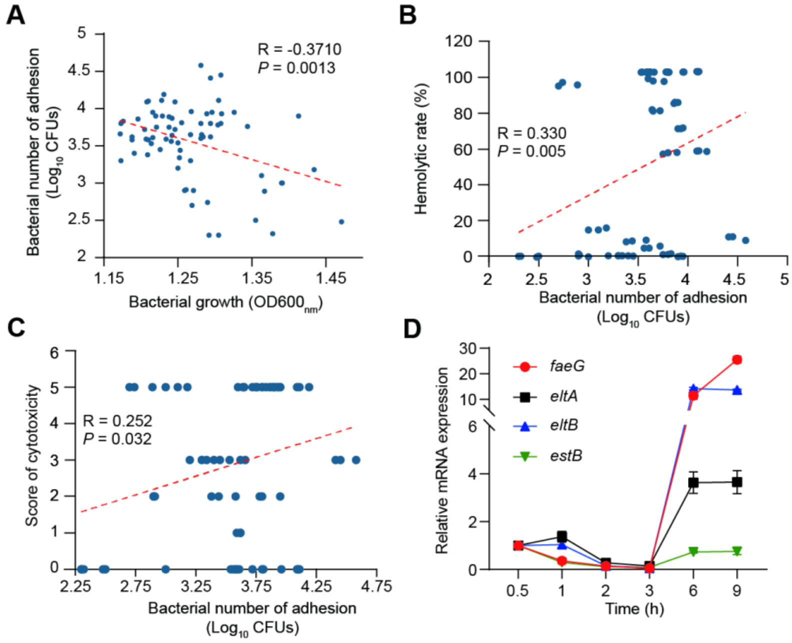

Figure S3 Correlation analysis among bacterial growth, adhesion and virulence, and expression of virulence genes

Table S4 Distribution of resistant genes among 24 ETEC from weaned pigs

ARGs	ETEC (n=24)	Fimbrial genotype			Serotype					
		K88 (n=7)	K99 (n=5)	F18ab (n=12)	O116:H11 (n=3)	O9:H19 (n=2)	O180:H9 (n=3)	O15:H18 (n=10)	O149:H10 (n=5)	O149:H9 (n=1)
**Aminoglycoside**										
aac(3)	19	7	0	12	3	2	0	9	5	0
aac(6′)	9	0	0	9	0	0	0	9	0	0
aac(3)-aac(6′)	13	1	0	12	3	1	0	9	0	0
aph(3′)	7	0	4	3	3	0	3	1	0	0
aadA	18	6	0	12	3	1	0	9	5	0
strA	4	0	4	0	0	0	3	1	0	0
strB	4	0	4	0	0	0	3	1	0	0
**β-lactam**										
blaZEG	16	7	5	8	3	2	3	2	5	1
blaNDM	6	5	1	0	0	0	0	1	5	0
blaTEM	2	1	0	1	0	1	0	1	0	0
blaCTX	1	1	0	0	0	1	0	0	0	0
blaACT	5	0	5	0	0	0	3	1	0	1

**Macrolide**										
mph(A)	14	6	0	8	0	1	0	8	5	0
mph(D)	14	6	0	8	0	2	0	8	4	0
mph(E)	5	5	0	0	0	0	0	0	5	0
msr(E)	5	5	0	0	0	0	0	0	5	0
mef(B)	9	6	0	3	3	1	0	0	5	0
erm(D)	1	1	0	0	0	0	0	0	1	0
**Quinolone**										
qnrB	8	0	0	8	0	0	0	8	0	0
qnrS	7	7	0	0	0	2	0	0	5	0
oqxB	24	7	5	12	3	2	3	10	5	1
**Florfenicol**										
floR	4	4	0	0	0	0	0	0	4	0
**Tetracycline**										
tet(A)	12	5	0	7	3	1	0	4	4	0
tet(B)	8	4	4	0	0	0	3	1	4	0
tet(M)	1	1	0	0	0	1	0	0	0	0

tet(S)	1	1	0	0	0	1	0	0	0	0
**Rifampin**										
arr-2	9	0	0	9	0	0	0	9	0	0
arr-3	18	6	0	12	3	1	0	9	5	0
arr-6	9	0	0	9	0	0	0	9	0	0
**Sulfonamide**										
dfrA	18	6	0	12	3	1	0	9	5	0
sul1	10	5	0	5	0	0	0	5	5	0
sul2	14	5	4	5	0	0	3	6	5	0
sul3	15	7	0	8	3	2	0	5	5	0
**Chloramphenicol**										
cml	5	2	0	3	3	2	0	0	0	0
cmlA1	4	1	0	3	3	1	0	0	0	0
